# Application-Oriented Bulk Cryopreservation of Human iPSCs in Cryo Bags Followed by Direct Inoculation in Scalable Suspension Bioreactors for Expansion and Neural Differentiation

**DOI:** 10.3390/cells12141914

**Published:** 2023-07-22

**Authors:** Ina Meiser, Monica Alstrup, Elham Khalesi, Bianca Stephan, Anna M. Speicher, Julia Majer, Chee Keong Kwok, Julia C. Neubauer, Mattias Hansson, Heiko Zimmermann

**Affiliations:** 1 Fraunhofer Institute for Biomedical Engineering (IBMT), Joseph-von-Fraunhofer-Weg 1, 66280 Sulzbach, Germany; ina.meiser@ibmt.fraunhofer.de (I.M.); bianca.stephan@ibmt.fraunhofer.de (B.S.); anna.speicher@ibmt.fraunhofer.de (A.M.S.); julia.majer@ibmt.fraunhofer.de (J.M.); julia.neubauer@ibmt.fraunhofer.de (J.C.N.); 2 Cell Therapy R&D, Novo Nordisk A/S, Novo Nordisk Park 1, 2760 Maaloev, Denmark; qmau@novonordisk.com (M.A.); eqkh@novonordisk.com (E.K.); czkw@novonordisk.com (C.K.K.); mshs@novonordisk.com (M.H.); 3 Department of Molecular and Cellular Biotechnology, Saarland University, 66123 Saarbruecken, Germany; 4 Facultad de Ciencias del Mar, Universidad Católica del Norte, Coquimbo 1781421, Chile

**Keywords:** hiPSC, cryopreservation, suspension bioreactors, cell therapy, bulk, expansion, differentiation, neurons, cryo bag

## Abstract

Stem cell-based therapies are promising tools for regenerative medicine and require bulk numbers of high-quality cells. Currently, cells are produced on demand and have a limited shelf-life as conventional cryopreservation is primarily designed for stock keeping. We present a study on bulk cryopreservation of the human iPSC lines UKKi011-A and BIONi010-C-41. By increasing cell concentration and volume, compared to conventional cryopreservation routines in cryo vials, one billion cells were frozen in 50 mL cryo bags. Upon thawing, the cells were immediately seeded in scalable suspension-based bioreactors for expansion to assess the stemness maintenance and for neural differentiation to assess their differentiation potential on the gene and protein levels. Both the conventional and bulk cryo approach show comparative results regarding viability and aggregation upon thawing and bioreactor inoculation. Reduced performance compared to the non-frozen control was compensated within 3 days regarding biomass yield. Stemness was maintained upon thawing in expansion. In neural differentiation, a delay of the neural marker expression on day 4 was compensated at day 9. We conclude that cryopreservation in cryo bags, using high cell concentrations and volumes, does not alter the cells’ fate and is a suitable technology to avoid pre-cultivation and enable time- and cost-efficient therapeutic approaches with bulk cell numbers.

## 1. Introduction

The discovery of human-induced pluripotent stem cells (hiPSCs) has been a game changer for diagnostics and therapy [1,2,3]. With their potential to differentiate into specialized cell types of each germ layer, hiPSCs are a valuable tool in diagnostics and therapy [4]. They can even be derived from somatic patient cells exhibiting a disease-specific background for modelling or drug-screening purposes. Their importance is reflected in the existence of numerous national as well as international banking initiatives, such as the European Bank of induced pluripotent Stem Cells [5,6,7] or the HLA-haplobank at Japanese Center for hiPS Cell Research and Application [8].

Pharmaceutical research and drug screenings have been reported to require between 5 × 10^8^ and 2 × 10^9^ cells per batch, depending on the targeted application and the selected compound library [9]. For therapeutic applications, the required number of cells is constantly increasing as new therapeutic approaches are developed. Benchmarks for cell quantities per therapeutic application are defined, such as 1 × 10^9^ cells per patient for liver cell transplantation [10] or 1 to 2 × 10^9^ in vitro differentiated cardiomyocytes for the treatment of heart failure [11].

Consequently, the request for bulk numbers of hiPSC is constantly on the rise, boosting the development of expansion technologies. Besides a number of highly specialised bioreactor systems, such as hollow-fiber reactors [12] or vertical-wheel bioreactors [13], and sophisticated automation approaches [14,15], scalable bioreactors are a promising approach. For example, Sharma et al. and Kwok et al. report on the expansion of human stem cells in their pluripotent state [10,16], whereas Altmaier et al. and Badenes et al. report on the bulk differentiation of hiPSCs [17,18]. The highest yield of stem cells to date (36 × 10^6^ cells per mL) was achieved with a 10 to 500 mL rotating cell culture, which is characterised by low shear and efficient gas transfer [19,20]. Of course, in each case, the output depends on the number of cells that has been used at the start. With the knowledge that pluripotent hiPSCs have the capability for exponential and potentially unlimited proliferation, it is reasonable to carefully stockpile them as starting material for bulk approaches, either for further expansion or differentiation.

The only way to ensure this is to cryopreserve the cells and store them in cryogenic temperatures below −130 °C, which also allows flexibility in time, extends the product’s shelf life, and enables simplified logistics [21]. However, the standard cryopreservation approach for hiPSCs uses conventional slow-rate freezing in cryo vials containing 1 × 10^6^ to 5 × 10^6^ cells in 1 to 2 mL volume in total [22]. During this process, the cells are frozen using a slow, cell type-specific cooling rate and cryoprotective agents [23]. The optimal cooling rate is primarily dependent on biophysical parameters, which must be specifically defined for each cell type [24]. A recent best-practice paper recommended a cooling rate of 1 °C/min and a range of 1 to 2 × 10^6^ cells in 1 mL [25], which is sufficient for keeping stock but insufficient for achieving application-ready quantities. Such aliquot sizes fall short of the requirements for large-scale applications, highlighting the urgent need for improved methods to generate and cryopreserve substantial quantities of up to one billion undifferentiated hiPSCs.

In this manuscript, we address the critical need to cryopreserve bulk numbers, namely 1 × 10^9^ hiPSCs. For such high cell numbers and consequently large volumes, cryo bags are already in use to store, e.g., bone marrow and peripheral blood stem cells [26,27]. Following the successful generation of the necessary number of starting hiPSCs in bioreactors and stockpiling them while maintaining quality, the translation into clinically applicable production designs will be enabled. Therefore, we paid attention to reproducibility, which is why we conducted a dual-side study. By using cryo bags, we overcome the limitations of the current standard routine and allow for stock keeping and immediate inoculation of the thawed cells in suspension-based bioreactors. Our study not only focuses on the quantity of cells but also emphasizes their quality and readiness for application. By implementing this innovative bulk cryopreservation strategy, we contribute to the advancement of hiPSC-based technologies, facilitating progress in both research and therapeutic fields.

## 2. Materials and Methods

### 2.1. hiPSC Culture

Human iPSC lines were obtained from EBiSC (www.ebisc.org, accessed on 14 June 2023; UKKi011-A; BIONi010-C-41) and were grown on cell culture dishes (Corning, New York, NY, USA) coated with 0.01 mg Matrigel per cm^2^ in mTeSR^TM^1 medium (Stemcell Technologies, Vancouver, BC, Canada) containing 1% Penicillin-Streptomycin-Glutamin (Gibco/Life Technologies, Carlsbad, CA, USA). For cryopreservation, the cells were expanded on 150 mm Matrigel™-coated plates in 2D over the course of 3 weeks. After expansion in 2D, the cells were inoculated in a CERO 3D bioreactor (OMNI Life Sciences, Bremen, Germany) at 1.25 × 10^6^ cells/mL in a total volume of 30 mL. The CERO 3D providing 37 °C and 5% CO_2_ and a rotation speed of 60 rpm was applied with a change of rotation orientation every 2 s. In the first 24 h of cultivation, the medium was supplemented with 10 µM Y-27632. After 24 h, for the quality controls, 15 mL of the cell suspension was taken from the CERO 3D tube, and the spheroids were dissociated using the Embryoid Body Dissociation Kit (Miltenyi Biotec, Bergisch Gladbach, Germany) according to the manufacturer’s instructions. Novo Nordisk used TrypLE^TM^ (Gibco) for dissociation of the spheroids. Two-thirds of the medium was changed daily. More details in Appendix A, Table A1.

### 2.2. Neural Differentiation

The EBiSC cell line BIONi010-C-41 is a gene-edited line containing NGN2 for transient, doxycycline- (DOX) inducible transgene expression facilitating differentiation into cortical neurons [28,29]. Inoculation of the cells (on day 0) using the CERO 3D bioreactor was performed in comparison to hiPSC culture conditions, with differentiation to neural fate initiated by DOX-induction on day 2. The DOX medium consists of 250 mL Neurobasal Medium (Gibco), 250 mL DMEM/F-12 (Gibco), GlutaMAX supplement (Gibco), 500 µL Pen/Strep/L-Glut (Gibco), 5 mL 50X B-27 Supplement (Gibco), 2.5 mL 100X N-2 Supplement (Gibco), 2.5 mL 100X MEM Non-EAAS (Gibco), 0.5 mM Sodium Pyruvate (Gibco), 2.5 mL 100X GlutaMAX Supplement (Gibco), 25 µM 2-Mercaptoethanol (Gibco), 2.85 µg/mL human insulin solution (Sigma, Darmstadt, Germany) and 20 µg/mL Doxycycline (Stemgent, Beltsville, MD, USA). Cultivation was continued until day 4 with a daily medium exchange of 66% using the DOX medium. Spheroids were then harvested and dissociated using the Embryoid Body Dissociation Kit according to the manufacturer’s instructions (Miltenyi Biotec, Bergisch Gladbach, Germany). The single cells were further cultivated at a concentration of 1 × 10^6^ cells/cm^2^ for 5 days on Matrigel™-coated dishes.

### 2.3. Cryopreservation Procedures

The effect of cryopreservation has been comparatively examined in two formats. Cryo vials (internal thread, 2 mL, Greiner bio-one) were used as standard cryo control, and 50 mL cryo bags (Miltenyi) were used for the bulk approach. For cryo vials: hiPSC were dissociated into single cells with TrypLE^TM^ (Gibco) for approx. 3 min at 37 °C. TrypLE^TM^ was removed, and the cells were rinsed with culture medium, collected, and centrifuged at 500× *g* for 3 min. Then, 2 × 10^7^ cells were taken up in 1 mL cryomedium CryoStor^®^ CS10 (Stemcell Technologies) containing 10% DMSO. The solution was transferred into a 2 mL cryo vial and cooled at −1 °C/min from 4 °C to −80 °C and then stored in the nitrogen tank for at least 2 days (Cryotherm, Kirchen/Sieg, Germany). The storage time allowed a simulation of the handling procedure, and longer storage periods were not considered necessary as the cell characteristics and functionality were not expected to be impaired since metabolic reactions come to a halt at these temperatures (Yannas 1968, Shafa 2019). (For cryo bags: 1 × 10^9^ cells were dissociated as stated before, resuspended in 50 mL CryoStor^®^ CS10 and added to a 50 mL cryo bag (Miltenyi Biotec) using a syringe. A cooling rate of –1 °C/min from 4 °C to −80 °C was conducted using an ASKION workbench (C Line^®^, WB220) and afterwards the cryo bag was stored in the nitrogen tank for at least 2 days.)

### 2.4. Thawing Procedures

The cryo vial and cryo bag were removed from the nitrogen tank and transferred to a 37 °C water bath (Julabo TW12). The cryo bag thawed at Novo Nordisk was shipped with a DryShipper (Chart DryShipper IATA XC, MVE), conditioned according to manufacturer’s instructions. The temperature was recorded during transport (ELPRO Ecolog TP2) and was below −160 °C. The cryo bag was taken from the DryShipper at Novo Nordisk and transferred to a 37 °C bead bath (Lab armor, Dallas, TX, USA) for thawing. Transportation using the DryShipper prevented multiple freeze-thaw cycles. The cell suspension was thawed until a small ice crystal was left (cryo vial approx. 1 min; cryo bag approx. 4 min). Afterwards the cell suspension was diluted 1:10 with DMEM F-12 medium (+ L-glutamine/+ 15 mM HEPES, Gibco) at RT and centrifuged at 500× *g* for 3 min. Then the cells were resuspended in mTeSR^TM^1 medium and further prepared for analysis and subsequent inoculation and cultivation in the suspension-based CERO 3D bioreactor (see Section 2.1 and Section 2.2).

### 2.5. Cell Counting, Viability, and Aggregation Rate Assessment

The NucleoCounter^®^ NC-200™ (Chemometec, Allerod, Denmark) was used to determine the cell count and viability of the cells. The hiPSCs were treated with lysis buffer A100 and stabilization buffer B (Chemometec, Allerod, Denmark) and measured in duplicates with the hiPSC-specific program of the instrument. The method makes use of the dyes acridine orange and 4′,6-diamidine-2-phenylindole (DAPI). The NucleoCounter^®^ software (NucleoView^TM^, Chemometec, Allerod, Denmark) provides the cell count [cells/mL] and viability [%]. To calculate the adhesion or aggregation rate, the cells were dissociated after 24 h cultivation in the respective regime (standard 2D cultivation or suspension-based 3D cultivation), centrifuged at 500× *g* for 3 min and recorded in a defined volume of cultivation medium. Subsequently, the live cell count was determined using the NucleoCounter^®^ NC-200™. For a percentage representation of the adhesion or aggregation rate, the resulting live cell count after 24 h was normalised to the cell count used for inoculation.

### 2.6. Quantitative Real-Time Polymerase Chain Reaction (qRT-PCR)

The RNA isolation was performed via the RNeasy Micro Kit (Qiagen, Hilden, Germany), following manufacturer’s instructions. A total of 250 ng of the isolated RNA was taken to perform a reverse transcription with High-Capacity cDNA Reverse Transcription Kit (Thermo Fisher Scientific, Waltham, MA, USA). Next, 0.2 µL cDNA was diluted in 4.3 µL RNA-free water and transferred in a 96-well plate with 5 μL TaqMan Fast Advanced Master Mix and 0.5 μL of the specific TaqMan Assay (both Thermo Fisher Scientific). A detailed list of primers (all Life Technologies, Carlsbad, CA, USA) is reported in Appendix A, Table A2. The qRT-PCR was conducted with the device QuantStudio 7 Flex and analysed with QuantStudio Real-Time software (Version 1.7.2, ThermoFisher Scientific, Waltham, MA, USA). Data were analysed with the Delta Delta Ct method, and the log fold change (relative) was plotted with hiPSCs as reference sample. *GAPDH* and *HPRT1* were used as endogenous controls.

### 2.7. Immunocytochemical Staining (ICC)

The cells were fixed for 30 min in BD Cytofix (BD Biosciences, Franklin Lakes, NJ, USA). The permeabilization was conducted using 0.2% Triton X in PBS for 20 min. Blocking of the unspecific bindings was done with 1% BSA and 0.2% Tween80 in PBS (blocking solution). The cells were incubated with primary antibodies diluted in the blocking solution (all BioLegend^®^, San Diego, CA, USA; types and concentrations shown in Appendix A, Table A3) overnight at 4 °C. Unattached antibodies were removed by washing 3 times for 10 min with blocking solution. Then the secondary antibodies diluted in the blocking solution were applied (all Thermo Fisher Scientific, types and concentrations shown in Appendix A, Table A4) and incubated for 1 h in the dark at room temperature. The nuclei were counterstained with DAPI (NucBlue™ ReadyProbes™ from Thermo Fisher Scientific). The images were recorded using the Confocal Microscope Leica TCS-SP8 Serial 8.

### 2.8. Flow Cytometry (FCM)

The cells were prepared for FCM by dissociation with TrypLE^TM^ (Gibco) and a washing step using PBS without calcium and magnesium. Fixation of the cells was conducted using BD Cytofix (BD Biosciences) for 30 min. The cells were washed with 5 mL PBS and then twice in 1 mL BD Perm/Wash Buffer III (BD Biosciences). Subsequently, the cells were resuspended in 100 μL FCM buffer (2% fetal bovine serum (FBS), 0.5 mM EDTA and 0.5 mM NaN3 in PBS). The cells were incubated with the antibodies (Appendix A, Table A5) for 30 min at 4 °C in the dark. The measurement was conducted with the cytometer Canto^TM^ II (BD Biosciences), and unstained cells served as the negative control if not stated otherwise.

### 2.9. Statistical Analysis

To determine significance, an appropriate Student’s *t*-test was performed using Origin (OriginLab, Version 2021, Northampton, MA, USA). Significance was determined at level *p* < 0.001 unless otherwise stated. Samples for cell functional analysis were performed in technological and biological triplicates unless indicated differently.

## 3. Results

### 3.1. Direct Inoculation of a Suspension Bioreactor with Cells Frozen in a Bulk Cryopreservation Approach

**Figure 1:**
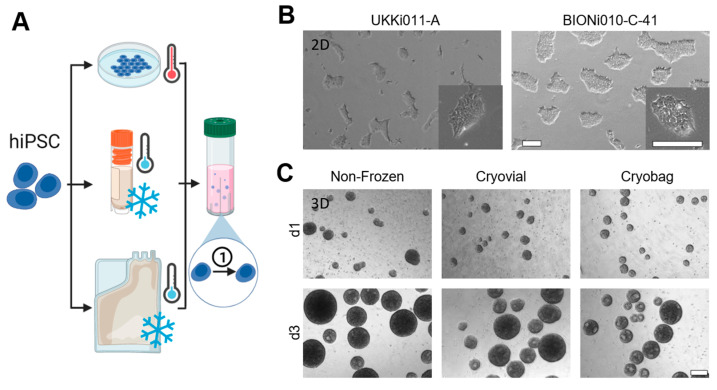
Morphology of UKKi011-A and BIONi010-C-41 cells before and after inoculation in scalable bioreactors. (**A**) Condensed schematic illustration of the workflow. The hiPSC lines UKKi011-A and BIONi010-C-41 were cryopreserved at a density of 2 × 10^7^ cells/mL using slow-rate freezing in cryo vials (1 mL, 2 × 10^7^ cells in total) and cryo bags (50 mL, 1 × 10^9^ cells in total) and directly inoculated in a CERO 3D-suspension bioreactor after thawing (➀ = stemness maintenance). (**B**) Phase contrast images showing the morphology of both cell lines before cryopreservation in 2D. Scale bars indicate 200 µm. (**C**) Representative phase contrast images of UKKi-011-A spheroids generated in a suspension-based bioreactor on day 1 and day 3 for the non-frozen samples, and the cryopreserved samples in cryo vials and cryo bags on day 1 and 3 after thawing. Scale bars indicate 500 µm.

In this study, hiPSC were cryopreserved in bulk quantity and subsequently inoculated in a single cell suspension in a CERO 3D bioreactor. The focus of investigation was on the retention of the pluripotency of the cells during 3-day cultivation. A schematic illustration of the condensed workflow is shown in Figure 1A. We compared non-frozen cells as a control with cells frozen in a standard cryo vial at a cell concentration of 2 × 10^7^ cells/mL and cells frozen at the same concentration in a 50 mL cryo bag, resulting in 1 × 10^9^ frozen cells. Both hiPSC cell lines, UKKi011-A and BIONi010-C-41, used in the experiments exhibited stem cell-like morphology (Figure 1B) with a dense packaging of small cells in colonies. The inoculation of the CERO 3D suspension bioreactor was conducted immediately after thawing to imitate an application-oriented workflow. Spheroids were formed in the course of day 1 and increased in size along the cultivation (Figure 1C). Cells inoculated immediately after thawing showed a more homogeneous spheroid size. At the end of the chosen cultivation period (3 days), the spheroids showed the formation of furrows occurring in all conditions and sizes (Figure 1C).

### 3.2. Performance of Bulk-Cryopreserved Cells in Bags Regarding Cultivation Parameters

**Figure 2:**
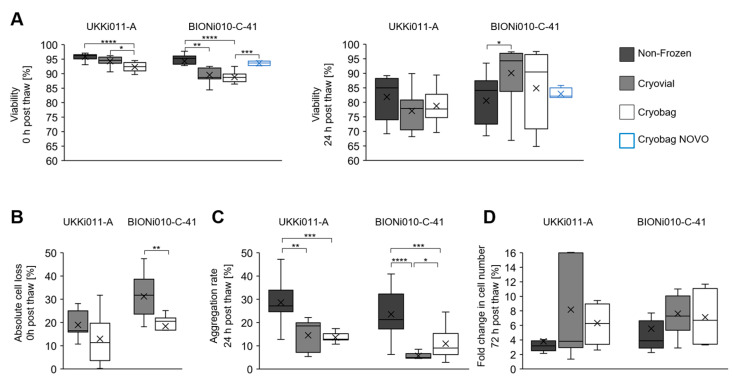
Viability, cell loss, aggregation rate, and fold change for UKKi011-A and BIONi010-C-41 after thawing in comparison to the non-frozen control. (**A**) Viability 0 and 24 h post-thaw of cells frozen in cryo vial (grey) and cryo bag (white) compared to the non-frozen control (black). In the scope of a two-centre study, Novo Nordisk provided viability results of cells frozen in cryo bag (blue). (**B**) Absolute cell loss 0 h post-thaw comparing cryo vial and cryo bag. (**C**) Aggregation rate 24 h post-thaw of non-frozen cells compared to cells frozen in cryo vial or cryo bag. (**D**) The fold change in cell number was calculated at 72 h post-thaw. The values were normalised to the cell number after 24 h after inoculation. Statistical significance was determined using a Student’s *t*-test (* *p* < 0.05; ** *p* < 0.01; *** *p* < 0.001; **** *p* < 0.0001).

The cells frozen in a cryo vial and cryo bag were compared to non-frozen control cells for the parameters viability, absolute cell loss, aggregation rate, and fold change (Figure 2). For UKKi011-A cells, the overall difference in viability directly after thawing between non-frozen and frozen samples was expressed in a statistically significant decrease of 3% from 96 to 93% showing minor standard deviations (Figure 2A). Concerning viability 24 h after thawing, values ranged between 70 and 97% with an increased standard deviation compared to 0 h post-thaw. For UKKi011-A, no significant differences were evident between the conditions. The absolute cell loss 0 h post-thaw was normalised to cells before the freezing process and shown for the frozen samples in Figure 2B. For both samples of the cell line UKKi011-A, the absolute cell loss was below 32%, and no statistically significant differences were observed. The aggregation rate of single cells to spheroids was determined 24 h after inoculation in the CERO 3D suspension bioreactor and was best in non-frozen cells with 29% (Figure 2C). In UKKi011-A there was no statistically significant difference between the frozen samples with 14% aggregation in average. The fold change of the thawed cells, determined 72 h after inoculation and normalised to the cell number 24 h after inoculation (Figure 2D), exceeded the non-frozen control. The cultivation parameters of the cell line BIONi010-C-41 show comparable results to UKKi011-A, with some evident cell-line-specific differences. The viability data for cells of the line BIONi010-C-41 frozen in the cryo bag were supported by values provided by Novo Nordisk as technical triplicates (Maaloev, Denmark) in the scope of a two-centre study. A statistically significant decrease in viability directly after thawing, compared to the control, of 6% to 89%, was observed to the same extent in both cryopreservation conditions (Figure 2A). The results generated for the cryo bag, frozen at Fraunhofer IBMT and then shipped and thawed at Novo Nordisk, showed a statistically significant higher viability 0 h post-thaw compared to the values collected at IBMT, reaching values of 94%. The viability 24 h post-thaw in cells frozen in cryo vials was 90%, significantly higher than the control (by 10%). The values for the bulk cryopreserved cells were characterised by a high standard deviation of 13%, but no significant differences from the other conditions were observed. The cell loss 0 h post-thaw of BIONi010-C-41 was 31% in cells cryopreserved in cryo vials and showed a significant decrease of 13% in the bulk cryopreservation approach (Figure 2B). The mean aggregation rate 24 h after thawing ranged from 6 to 24%, with non-frozen cells showing 24% (Figure 2C). The bulk cryopreserved cells in cryo bags were significantly better than the ones cryopreserved in vials, according to the standard cryo protocol, with a 5% increase in aggregation to 11%. The fold change in cell number 72 h post-thaw was comparable between BIONi010-C-41 and UKKi011-A.

### 3.3. Performance of Bulk-Cryopreserved Cells in Bags Regarding Maintenanceof Pluripotency Characteristics

**Figure 3:**
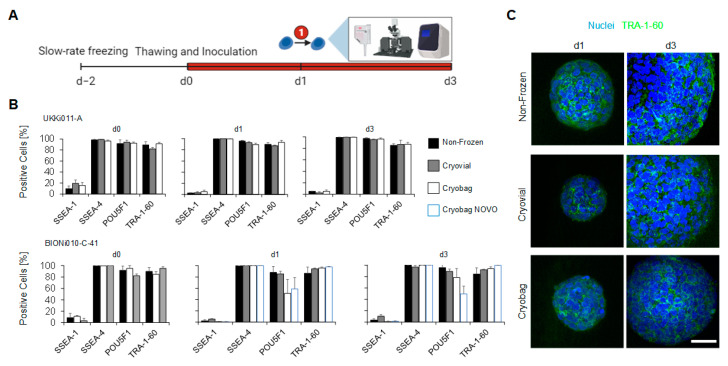
Analysis for stemness maintenance on protein level via FCM and ICC. (**A**) Timeline of the experiment. Cells were frozen in either cryo bags or vials and inoculated in a suspension bioreactor upon thawing. Samples were taken at 0, 1, and 3 days for analysis of pluripotency (➀ = stemness maintenance). (**B**) FCM studies were performed using the markers SSEA-1, SEA 4, POU5F1, and TRA-1-60. (**C**) Representative fluorescence microscopy images of UKKi011-A spheroids verified pluripotency-marker TRA-1-60 expression. Scale bars indicate 50 µm.

At different time points of the post-thaw cultivation in suspension bioreactors, the cells were analysed for pluripotency markers via FCM, ICC and qRT-PCR (Figure 3A). The pluripotency maintenance during the course of 3 days was verified at protein level, using the markers SSEA-1 (negative control), SSEA-4, POU5F1, and TRA-1-60, as shown in Figure 3B. According to guidelines of the European Bank for induced pluripotent Stem Cells (EBiSC.org), a maximum of 10% is allowed to be SSEA-1 positive, and a minimum of 70% of the cells must be SSEA-4, POU5F1 and TRA-1-60 positive to qualify as stem cell population. UKKi011-A met this criterion to a full extent; however, a decrease of the marker POU5F1 to 50% in the cell line BIONi010-C-41 frozen in a cryo bag was observed after 1 day of cultivation. This decrease was compensated after 3 days of cultivation in the suspension bioreactor, when 78% of the cells were POU5F1-positive. Data collected at Novo Nordisk remained at 50% for POU5F1 for this cell line. A visual verification of the expression and localisation of the glycoprotein TRA-1-60 was performed via ICC staining (Figure 3C). The protein TRA-1-60 was distributed homogeneously throughout the spheroid.

**Figure 4:**
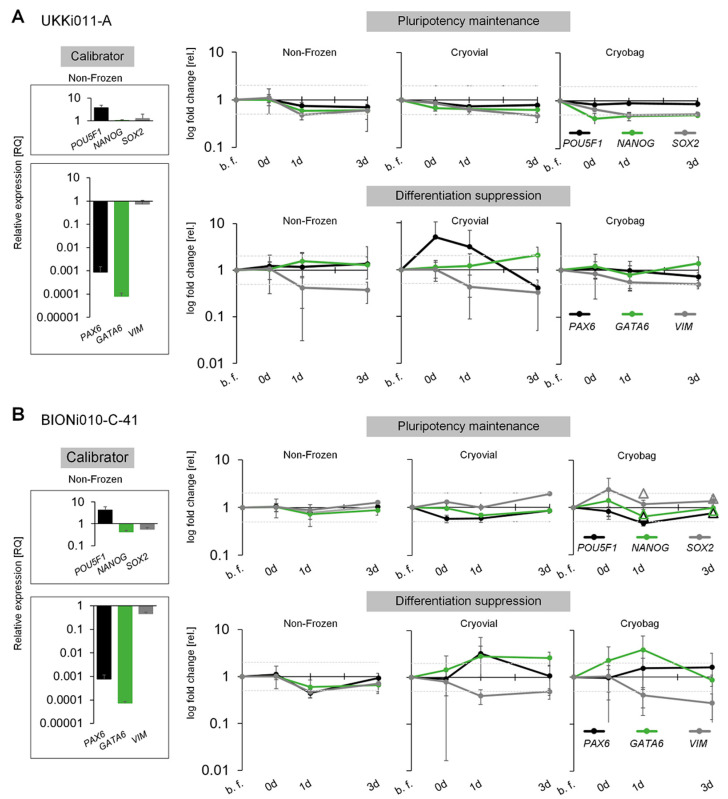
Analysis for stemness maintenance on a genetic level via qRT-PCR. (**A**) Pluripotency markers *POU5F1* (black), *NANOG* (green) and *SOX2* (grey), as well as the specific markers *PAX6* (ectoderm specific, black), *GATA6* (endoderm specific, green), *VIM* (mesoderm specific, grey) were analysed during a 3-day cultivation for the cell line UKKi011-A. (**B**) The same analysis was conducted for the cell line BIONi010 C 41. The results of Novo Nordisk, complementing the ones of Fraunhofer IBMT, concerning the cells of the cryo bag are depicted as triangles in the same colour. The relative expression values for the used calibrator (non-frozen control at time point before freezing (b. f.)) are shown on the left of each panel. All values are normalised to the house keeping genes *GAPDH* and *HPRT1*.

The maintenance of pluripotency in both cell lines UKKi011-A and BIONi010-C-41 was additionally verified on a genetic level throughout the expansion via qRT-PCR. All values are normalised to the house-keeping genes *GAPDH* and *HPRT1*. The expression of stem cell markers *POU5F1*, *NANOG*, and *SOX2* as well as the ectoderm marker *PAX6,* endoderm marker *GATA6*, and mesoderm marker *VIM* were examined (Figure 4). The relative change in gene expression was calibrated to the non-frozen control at the beginning of cultivation. The high expression level of *POU5F1* and the low level of the differentiation markers (Figure 4A,B), indicate the pluripotent stem-cell fate of the cells at the start of inoculation on day 0. For UKKi011-A, the expression of the pluripotency genes *POU5F1*, *NANOG*, and *SOX2* remained stable in all conditions over the course of expansion (Figure 4A). Also, no major changes in gene expression were observed for endodermal *GATA6*. However, cells frozen in standard cryo vials showed a 12-fold upregulation in ectodermal *PAX6* directly after thawing, which decreased again along the cultivation duration. The mesodermal *VIM* expression in UKKi011-A showed a statistically significant down-regulation until day 3 of the cultivation in all conditions, but with a rather small relative fold change of 2. The cell line BIONi010-C-41 depicted similar results; the pluripotency markers stayed stable in all conditions (Figure 4B). Novo Nordisk again supplemented the gene expression data for the stem cell markers for cells frozen in cryo bags and achieved comparable results (shown as triangles in Figure 4B). For the ectodermal marker *PAX6,* a significant fold change of 3 was observed in cells frozen in cryo vials, which levelled until day 3 of cultivation. The endodermal marker *GATA6* was upregulated in cells from the cryo vial on day 1 but returned to baseline on day 3. A downregulation of the mesoderm marker *VIM* was evident in cells frozen in the cryo bag. All measured marker expressions indicate successful differentiation suppression.

### 3.4. Performance of Bulk-Cryopreserved Cells in Bags, Regarding Their Differentiation Capacity

**Figure 5:**
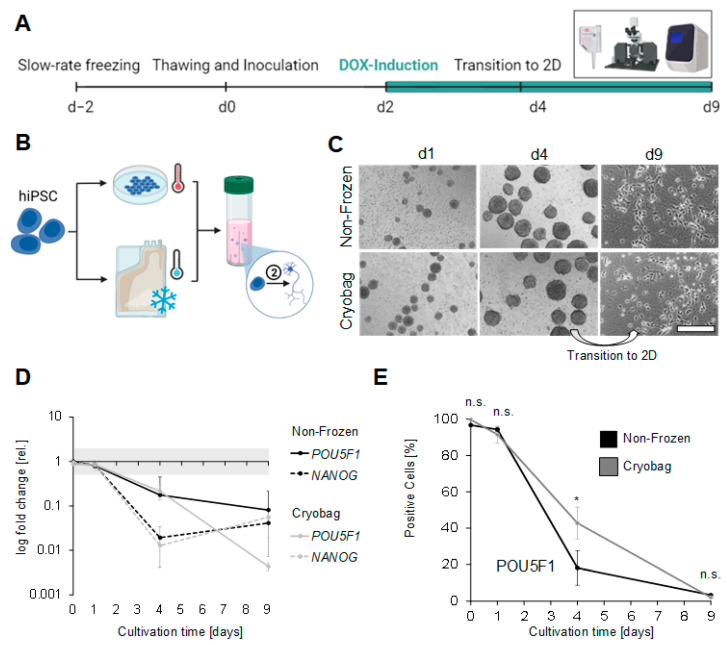
Differentiation potential of BIONi010-C-41 after bulk cryopreservation. (**A**) Timeline of the experiment. (**B**) Condensed schematic illustration of the workflow. BIONi010-C-41 cells were cryopreserved in a cryo bag (50 mL with 1 × 10^9^ cells in total) using slow-rate freezing and, after thawing, directly inoculated in a suspension-based bioreactor (➁ = neural differentiation). (**C**) Phase-contrast images showing the spheroid’s morphology at day 1 and day 4, as well as the morphology of mature neurons after transition to 2D at day 9 of cultivation. Scale bars indicate 500 µm. (**D**) qRT PCR analysis of pluripotency markers (*POU5F1, NANOG*) for non-frozen (black) and bulk-frozen cells after thawing (grey). (**E**) FCM analysis of pluripotency markers POU5F1 comparing non-frozen (black) and bulk cryopreserved cells (grey). Statistical analysis was performed using a Student’s *t*-test (n.s. = not significant; * *p* < 0.05).

The gene-edited cell line BIONi010-C-41 was examined for its capacity to differentiate into mature neurons after cryopreservation (Figure 5A). Non-frozen and thawed cells of this line were therefore inoculated in suspension-based bioreactors and differentiated into neural cells using DOX-inducible NGN2 overexpression, starting at day 2 of cultivation (Figure 5B). To generate mature neurons, the spheroids were differentiated for 2 additional days in the bioreactor before they were dissociated at day 4 and plated on Matrigel™-coated dishes for a further 5 days of cultivation in 2D. Representative microscopic images were taken, showing smooth spheroid surfaces on day 1 that got frayed along the course of the cultivation for both conditions (Figure 5C). The cell-free cysts that appeared in hiPSC spheroids did not occur in the differentiation. After transition to 2D and at the end of cultivation, the neurons showed a homogeneous distribution and characteristic neural morphology with long and branched axons (Figure 5C). There were no morphological differences between the non-frozen control and frozen cells. Additionally, the differentiation state was assessed by FCM and qRT-PCR on days 0, 1, 4, and 9. To verify a successful neural differentiation, the relative gene expression of the pluripotency markers *POU5F1* and *NANOG* was evaluated in comparison to non-frozen hiPSCs (Figure 5D). In both conditions (non-frozen and frozen), a down-regulation of both markers already 2 days after DOX induction indicated the loss of pluripotency. With continued DOX induction, *POU5F1* was further downregulated, with the formerly bulk-frozen cells showing a significantly stronger decrease. The gene expression of *NANOG* shows an analogous course for the non-frozen and frozen cells with a maximum decrease 4 days after induction. FCM was performed to investigate the stem cell marker POU5F1 on the protein level as well (Figure 5E). Before differentiation, more than 91% of all cells showed an expression of the stem cell marker POU5F1. The number of POU5F1-positive cells plummeted after 2 days of DOX induction, with a significantly faster decrease in the non-frozen cells. By day 9, hardly any POU5F1-positive cells were detectable.

**Figure 6:**
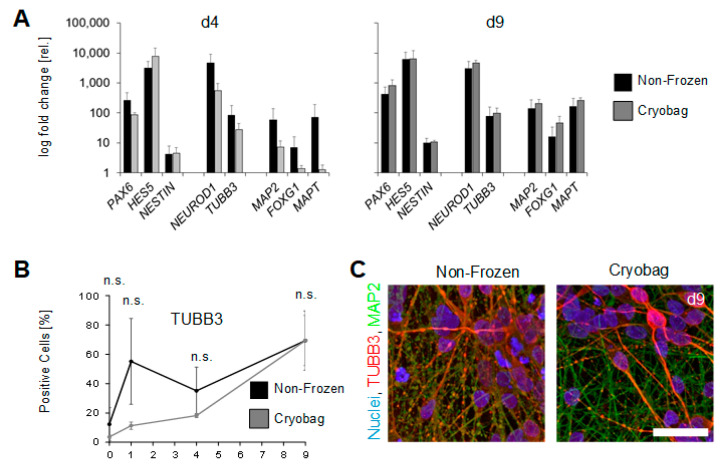
DOX-induced neural differentiation of BIONi010-C-41 after bulk cryopreservation. (**A**) qRT-PCR analysis of the BIONi010-C-41 along the differentiation after DOX induction into mature neurons. The ectoderm-specific markers *PAX6, HES5* and *NESTIN*, the markers for immature neurons *NEUROD1* and *TUBB3.* For mature neurons, *MAP2*, *FOXG1,* and *MAPT* were used as indicators of neural fate. In cryopreserved cells, a delayed expression of neural markers was observed, but at the end of differentiation, gene expression was comparable to the non-frozen control. (**B**) In a FACS analysis, the marker of immature neurons TUBB3 was examined on the protein level, indicating an increase during the cultivation in both conditions. Statistical analysis was performed using a Student’s *t*-test (n.s. = not significant). (**C**) Representative fluorescence microscopy images of neurons after 9 days of cultivation verify the expression and the location of immature neuron marker TUBB3 and mature neuron marker MAP2. Scale bars indicate 50 µm.

The state of neuronal differentiation was additionally analysed via qRT-PCR, using the ectoderm-specific markers *PAX6*, *HES5* and *NESTIN*, the marker for immature neurons *NEUROD1*, the early maturation marker *TUBB3,* and the late maturation markers *MAP2*, *FOXG1* and *MAPT* (Figure 6A). Starting from neural induction, an upregulation of neuron-specific markers was observed in all conditions, indicating the strike of neural fate. An evident delay of the gene expression of all analysed markers was observed in the formerly cryopreserved cells, that, however, seemed to be compensated at day 9 of the course of differentiation. Two days after DOX induction (on day 4), the expression of immature, early and late neural markers in the cryo bag samples lagged behind compared to the non-frozen control. On day 4, *NEUROD1* and *TUBB3* showed the highest expression in non-frozen cells. In the further course of differentiation of the cryopreserved cells, the gene expression level of all analysed markers was higher than the levels of the non-frozen control. In further experiments, BIONi010-C-41 was analysed during differentiation for protein expression, focusing on the early mature neuron marker TUBB3 (Figure 6B). Overall, TUBB3 protein expression was increased in the course of cultivation, with a maximum of 12% TUBB-positive cells at the beginning of cultivation. After 1 day in culture, before induction of neural differentiation, the number of TUBB-positive cells averaged 55% in the control group, with a large standard deviation. The protein-expression level of TUBB3 dropped until day 4 in the non-frozen cells to reach 70% of TUBB3-positive cells at the end of cultivation in both conditions. The markers TUBB3 and MAP2 were further investigated by ICC to determine their distribution and localisation within the cells (Figure 6C). In differentiated neurons at day 7 of DOX induction, MAP2 was delineated clearly in the dendrites and cytoskeleton of the cells in green. TUBB3 was localised in the cytoplasm of the cells and was also detected along the dendrites of the neurons.

## 4. Discussion

Human stem cell-based therapeutic products have already paved their way to clinical application and are on the rise. They are considered promising treatment for multiple human diseases, such as neurological or reproductive disorders, wound healing, or cardiovascular conditions [30]. Depending on the final application, of course, the demand on cell numbers per batch ranges from several millions to up to billions of cells per patient [11], leading the focus on sophisticated, scalable manufacturing processes [31] as well as comprehensive quality-control regimes for source cells and final products [20,32,33]. Regarding the demand for high cell numbers, suspension-based bioreactors are the technology of choice to produce a sufficient number of cells because they exhibit a much more preferable ratio of media consumption per cell number compared to conventional 2D cultivation technologies [34,35]. To start a production process on demand, a frozen bulk stock of stem cells ready to be seeded upon thawing would avoid pre-expansion with extensive quality controls along the cultivation process [36]. The idea of bulk storage has already been investigated in a crystallization-free vitrification approach. However, the handling and throughput in this still experimental cryo regime need to be optimized to meet prerequisites for GMP-compliant clinical processes [37,38]. Considering the conventional slow-freezing cryopreservation, established infrastructure compatible with GMP requirements is available, but these standard routines use small-volume aliquots not designed to meet the requested bulk cell numbers.

### 4.1. Pre-Studies Determined Cell Concentration and ROCK Inhibition

In pre-studies, we investigated a suitable cell concentration (from 1 × 10^6^ to 2 × 10^7^ cells/mL) in 1 mL in cryo vials first, before increasing the volume to 50 mL in cryo bags. We did not detect a statistically significant influence of the 10-fold increase of the cell concentration (viability 0 h after thawing was 90 ± 3% in biological and technical triplicates). The increase in cell number per volume did not show an effect on the freezing efficiency, leading to the assumption that crystallization under the applied conditions is still initiated in the extracellular medium in accordance with investigations from Hunt et al. [39]. Ice nucleation in the extracellular space leads to an increase in solutes and results in an osmotic gradient that helps the cells, in combination with a suitable cell-type-specific cooling rate, to dehydrate to a certain extent and to avoid intracellular ice formation [40]. Additionally, we checked the influence of ROCK inhibition in pre-studies, as ROCK inhibitors are known to increase survival rates in human stem cells after thawing [41] or after dissociation [42]. Whereas Li et al. found an increase of the survival rate of human embryonic stem cells after thawing from 5% to 53% [43], Miyazaki et al. found that an optimal seeding density can enhance the viability after thawing, similarly to the application of a ROCK inhibitor [44]. ROCK inhibitors influence signal cascades responsible for apoptosis [45], as well as for the regulation of the cytoskeleton or membrane proteins such as E-cadherin, relevant for aggregation of cells [46]. According to live cell imaging, the hiPSCs aggregate within the first hour after inoculation in static conditions. With the tested inoculation densities, the addition of ROCK inhibitor Y-27632 was necessary for aggregation, as has been reported before [47].

### 4.2. More Homogeneous Aggregates after Bulk Freezing in Bags

In the present study, aggregate sizes differ among the compared approaches (non-frozen control, standard cryo vial freezing and bulk freezing in cryo bags). Aggregates formed after bulk freezing show the smallest diameter, which can be explained by their reduced viability upon thawing. Less viable cells will spontaneously find each other and thus form smaller aggregation nuclei. The reduced viability can be a result of increased mechanical stress on bulk frozen cells in cryo bags because of more pipetting steps, since hiPSCs are known to be shear-stress sensitive [48]. Another explanation could be a less favourable freezing process resulting from the different material parameters of cryo bags and increased volume while other parameters, especially the applied cooling rate, remained unchanged: Cryo bags are composed of ethylene vinyl acetate, which has a thermal conductivity that is 5.6 times higher than polypropylene cryo vials [49]. Combined with the larger freezing volume, ice propagation, and thereby, setting a state of equilibrium starts earlier and takes longer, exposing the cells longer to a hyperosmolar medium. Mazur described the negative effect of a too-slow cooling rate in his Two-Factor-Hypothesis, claiming that solution effects due to forming ice crystals harm the cells via dehydration and structural damages [50]. Although cells frozen in the cryo bag present with a lower viability than cells frozen in cryo vials, the overall cell loss is higher in cryo vials, which might be attributed to cell loss during post-thaw processing, like pipetting and centrifuging, which have a greater percentage effect for small volumes compared to large volumes.

### 4.3. Maintenance of Stemness upon Thawing and Bioreactor Cultivation

We show that the original cell fate of both cell lines is not altered by an increased cell density and volume during cryopreservation. The hiPSCs maintain their stemness characteristics and meet the applied EBiSC quality-control criteria, proven by mRNA and protein expression. FCM and qRT-PCR data show no significant changes during cryopreservation and a cultivation period in suspension for 3 days, which figure among the approaches (non-frozen, frozen in cryo vials, and frozen in cryo bags) taken in a dual-centre study at Fraunhofer IBMT in Germany (production and evaluation site for UKKi011-A and BIONi010-C-41 in biological and technical triplicates) and Novo Nordisk in Denmark (evaluation site for UKKi011-A in technical triplicates). Additionally, the hiPSC lines were checked for differentiation suppression using the markers *PAX6, GATA6,* and *VIM*. While the UKKi011-A remained unsuspicious after 3 days of culture upon bulk-freezing at both sites (outliers post-thawing for *PAX6* in vials), BIONi010-C-41 showed an increase in *VIM* expression and a decrease in *PAX6* expression at Novo Nordisk. Results at Fraunhofer IBMT cannot confirm these findings, unlike the rest of the data. Thus, either the shipping process or minor differences in handling during and after thawing may have caused this aberration. That shows how cautiously hiPSCs need to be processed and handled, especially regarding a potential clinical application. The increased expression of *VIM* is most likely a stress response, unrelated to differentiation [51]. And as the BIONi010-C-41 is edited to ease neural differentiation, the genetic background might favour mesodermal fate.

### 4.4. Cell-Line Specific Differences in Performance

Our studies are also evidence of cell-line-specific differences when working with hiPSCs: Different genetic backgrounds and reprogramming routines lead to different performances of both cell lines when it comes to recovery 24 h after cryopreservation, as reported in other studies [52]. The differences are already obvious in the morphology of the standard 2D cultivation. However, for both cell lines investigated in this study, we found a reduced viability 0 h post-thaw and a reduced aggregation rate 24 h post-thaw. The initial cell loss resulting from the slow freezing process could be compensated after direct inoculation in suspension-based bioreactors by both cryo approaches compared to the non-frozen cells within 3 days. At the protein level, BIONi010-C-41 shows a significant decrease of POU5F1 after bulk freezing in cryo bags, which was documented at both sites and returned to normal after 3 days of cultivation. The same phenomenon has already been reported for human embryonic stem cells [53] and is most likely a result of cryo-induced cell stress e.g., via an increased amount of ROS [54,55] or membrane damage. This discrepancy between the cell lines indicates that BIONi010-C-41 is more susceptible to the increased mechanical forces due to cryo-bag handling. Additionally, it again could indicate that the edited gene line is in favour of differentiation, which, however, is levelled out along the expansion under standard conditions.

### 4.5. Neuronal Differentiation Capacity Maintained but Delayed

Stem cell therapies are particularly interesting for the treatment of neurodegenerative diseases such as Parkinson’s [56], which is why our study paid attention to the capability of the bulk frozen hiPSCs to differentiate into neurons. The maintained differentiation capacity after cryopreservation is shown for the BIONi010-C-41-line frozen in cryo bags. The forced overexpression of the transcription factor neurogenin-2 (NGN2) via DOX induction of BIONi010-C-41 started 2 days after inoculation in the bioreactor and enables a rapid production of neural cells within 7 days, which is highly desirable in terms of flexible time management [57]. The suppression of pluripotency markers was congruent among the non-frozen control and the bulk-frozen samples on mRNA and protein level along the differentiation process. However, qRT-PCR data revealed that the cryopreservation process leads to a delay in neuronal marker expression (*NEUROD1*, *TUBB3*, *MAP2*, *FOXG1*, *MAPT*). A TUBB3 peak documented via FCM at day 1 (before DOX induction) in the non-frozen control is unexpected and most likely an outlier, considering the standard deviations. In the bulk-frozen sample, no outlier was detected but might have appeared with a delay that was not included in our analysis schedule. TUBB3 is involved in axon guidance [58], and we verified its correct location via ICC staining, as with the mature neuron marker MAP2 in the final analysis (day 9) [59]. We hypothesize that the cryo-related delay in marker expression results from the impairment of cellular functions such as the regulation of transcription and protein synthesis through reduced energy metabolism, as previously shown in horse spermatozoa [60]. This effect probably manifests itself in a down-regulation of promoter or enzyme activity mandatory for NGN2 expression and following downstream processes of the neural differentiation. A reduced efficiency of DOX induction, which might result from a cryo-induced cellular stress response [61,62], would lead to a decreased NGN2 concentration in comparison to non-frozen cells and might thus explain the lower expression of the early neuronal marker NEUROD1, which is directly regulated by NGN2. All mature neuronal markers would consequently also show low expression profiles. As far as we can conclude in this study, the delay has no consequences on further development into neurons, as the marker expression does not differ from the non-frozen cells at the end point analysis on day 9.

## 5. Conclusions

We conclude that slow-rate bulk freezing of hiPSCs is successful and comparable to the conventional standard routine. The fate of cells is not altered when they are frozen with an increased cell concentration and volume (2 × 10^7^ cells/mL in 50 mL to reach a final cell number of 2 × 10^9^ cells per batch). The initial decrease in viability as well as the delay in marker expression during differentiation is compensated within days upon immediate seeding in suspension-based bioreactors without a pre-cultivation phase. To meet the needs of future stem cell-based therapies, application-oriented cryopreserved products have to be developed. To supply high cell numbers, cryo bags are a promising format, and our study showed their feasibility to provide high-quality cells. Depending on the final application, a further increase in cell concentration and volume per bag is conceivable, as well as an optimization of the inoculation protocol, which was not a scope of our study. The concept can also be applied to other cell systems, e.g., hiPSC-derived neural or cardiac progenitors. The bulk cryopreservation approach in bags leads to a decrease in consumables, such as cryo media, and subsequently to a reduction of storage space and costs.

## Data Availability

Data are contained within the article.

## Appendix A

The appendix contains detailed information on primers, antibodies and other consumables.

**Table A1:** List of Consumables.

Name	Cat. Nr.	Source
2-Mercaptoethanol	31350010	Gibco™
B-27 Supplement	17504044	Gibco™
BD Cytofix™ Fixation Buffer	554655	BD Biosciences
BD Perm/Wash Buffer	554723	BD Biosciences
Bovine serum albumin	0332-25G	VWR
CryoStor^®^ CS10	07930	Stemcell™ Technologies
DMEM/F-12 (+ L-glutamine/+ 15 mM HEPES)	11330057	Gibco™
DMEM/F-12, GlutaMAX™ supplement	31331028	Gibco™
Doxycycline	04-0016	Stemgent
EDTA	AM9260G	Invitrogen™
Embryoid Body Dissociation Kit	130-096-348	Miltenyi
Fetal bovine serum (FBS)	10500-064	Gibco™
GlutaMAX™ Supplement	35050038	Gibco™
High-Capacity cDNA Reverse Transcription Kit	4368814	Applied Biosystems™
human Insulin solution	I9278	Sigma-Aldrich
Lysis buffer A100	910-0010	Chemometec
Matrigel^®^	354230	Corning^®^
MEM Non-EAAS	11140035	Gibco™
mTeSR™1 medium	85850	Stemcell™ Technologies
N-2 Supplement	17502048	Gibco™
Neurobasal Medium	21103049	Gibco™
Penicillin-Streptomycin-Glutamin	10378016	Gibco™
Phosphate buffered saline	11540546	Gibco™
RNeasy Micro Kit	74004	Qiagen
Sodium azide (NaN3)	S2002	Sigma Aldrich
Sodium Pyruvate	11360070	Gibco™
Stabilisation buffer B	910-0002	Chemometec
TaqMan^®^ Fast Advanced Master Mix	4444557	Applied Biosystems™
Triton™ X-100	T878-100ML	Sigma-Aldrich
TrypLE™ Select	11598846	Gibco™
Tween^®^ 80	P4780-100ML	Sigma-Aldrich
Y-27632	10005583	Cayman Chemicals

**Table A2:** List of qRT-PCR primers.

Target	TaqMan Assay ID	Source
GAPDH	Hs99999905_m1	Life Tech, Thermo Fisher Scientific
HPRT1	Hs99999909_m1	
POU5F1	Hs00742896_s1	
NANOG	Hs04399610_g1	
SOX2	Hs00602736_s1	
PAX6	Hs01088114_m1	
GATA6	Hs00232018_m1	
VIM	Hs00185584_m1	
HES5	Hs01387463_g1	
NESTIN	Hs04187831_g1	
NEUROD1	Hs01922995_s1	
TUBB3	Hs00964962_g1	
MAP2	Hs00258900_m1	
FOXG1	Hs01850784_s1	
MAPT	Hs00902194_m1	

**Table A3:** List of ICC primary antibodies.

Antibody	Host Species	Isotype	Dilution	Cat. Nr.	Source
TRA-1-60-R	Mouse	IgM, κ	1:400	330602	BioLegend^®^
Tubulin β 3 (Clone: TUJ1)	Mouse	IgG2a, κ	1:400	801201	BioLegend^®^
MAP2	Rabbit	IgG	1:100	840601	BioLegend^®^

**Table A4:** List of ICC secondary antibodies.

Antibody	Host Species	Isotype	Fluorophor	Dilution	Cat. Nr.	Source
Anti-Mouse	Donkey	IgG H + L	Alexa Fluor^TM^ 488	1:10,000	A21202	Thermo Fisher Scientific
Anti-Rabbit	Goat	IgG	Alexa Fluor™ 594	2 drops/mL	R37117	Invitrogen

**Table A5:** List of FCM antibodies.

Antibody	Host Species	Isotype	Fluorophor	Dilution	Cat. Nr.	Source
Oct3/4	Mouse	IgG1, κ	PerCP-Cy^TM^5.5	1:5	560794	BD Biosciences
TRA-1-60-R	Mouse	IgM, κ	Alexa Fluor^®^ 647	1:20 (5µL in 100 µL staining volume)	330606	BioLegend^®^
CD15 SSEA-1	Mouse	IgM, κ	PE/Cyanine7	1:20	301924	BioLegend^®^
Isotype Ctrl	Mouse	IgM, κ	PE/Cyanine7	1:20	401628	BioLegend^®^
SSEA4	Human	-	FITC	1:5	130-098-371	Miltenyi Biotec
anti-β-Tubulin, Class III	Mouse	IgM, κ	Alexa Fluor^®^ 647	1:20	558606	BD Biosciences

## References

[1] Nishikawa S., Goldstein R.A., Nierras C.R. (2008). The promise of human induced pluripotent stem cells for research and therapy. Nat. Rev. Mol. Cell Biol..

[2] Parrotta E.I., Scalise S., Scaramuzzino L., Cuda G. (2019). Stem Cells: The Game Changers of Human Cardiac Disease Modelling and Regenerative Medicine. Int. J. Mol. Sci..

[3] Takahashi K., Yamanaka S. (2013). Induced pluripotent stem cells in medicine and biology. Development.

[4] Shi Y., Inoue H., Wu J.C., Yamanaka S. (2017). Induced pluripotent stem cell technology: A decade of progress. Nat. Rev. Drug Discov..

[5] Steeg R., Neubauer J.C., Müller S.C., Ebneth A., Zimmermann H. (2020). The EBiSC iPSC bank for disease studies. Stem Cell Res..

[6] de Sousa P.A., Steeg R., Wachter E., Bruce K., King J., Hoeve M., Khadun S., McConnachie G., Holder J., Kurtz A. (2017). Rapid establishment of the European Bank for induced Pluripotent Stem Cells (EBiSC)—The Hot Start experience. Stem Cell Res..

[7] Stacey G. (2017). Stem Cell Banking: A Global View. Methods Mol. Biol..

[8] Yoshida S., Kato T.M., Sato Y., Umekage M., Ichisaka T., Tsukahara M., Takasu N., Yamanaka S. (2023). A clinical-grade HLA haplobank of human induced pluripotent stem cells matching approximately 40% of the Japanese population. Med.

[9] Wrigley J.D., McCall E.J., Bannaghan C.L., Liggins L., Kendrick C., Griffen A., Hicks R., Fröderberg-Roth L. (2014). Cell banking for pharmaceutical research. Drug Discov. Today.

[10] Sharma S., Raju R., Sui S., Hu W.-S. (2011). Stem cell culture engineering—Process scale up and beyond. Biotechnol. J..

[11] Serra M., Brito C., Correia C., Alves P.M. (2012). Process engineering of human pluripotent stem cells for clinical application. Trends Biotechnol..

[12] Vymetalova L., Kucirkova T., Knopfova L., Pospisilova V., Kasko T., Lejdarova H., Makaturova E., Kuglik P., Oralova V., Matalova E. (2020). Large-Scale Automated Hollow-Fiber Bioreactor Expansion of Umbilical Cord-Derived Human Mesenchymal Stromal Cells for Neurological Disorders. Neurochem. Res..

[13] Borys B.S., Dang T., So T., Rohani L., Revay T., Walsh T., Thompson M., Argiropoulos B., Rancourt D.E., Jung S. (2021). Overcoming bioprocess bottlenecks in the large-scale expansion of high-quality hiPSC aggregates in vertical-wheel stirred suspension bioreactors. Stem Cell Res. Ther..

[14] Davis B.M., Loghin E.R., Conway K.R., Zhang X. (2018). Automated Closed-System Expansion of Pluripotent Stem Cell Aggregates in a Rocking-Motion Bioreactor. SLAS Technol..

[15] Elanzew A., Nießing B., Langendoerfer D., Rippel O., Piotrowski T., Schenk F., Kulik M., Peitz M., Breitkreuz Y., Jung S. (2020). The StemCellFactory: A Modular System Integration for Automated Generation and Expansion of Human Induced Pluripotent Stem Cells. Front. Bioeng. Biotechnol..

[16] Kwok C.K., Sébastien I., Hariharan K., Meiser I., Wihan J., Altmaier S., Karnatz I., Bauer D., Fischer B., Feile A. (2022). Scalable expansion of iPSC and their derivatives across multiple lineages. Reprod. Toxicol..

[17] Altmaier S., Meiser I., Lemesre E., Chanrion B., Steeg R., Leonte L.E., Holst B., Nielsen B.S., Clausen C., Schmidt K. (2022). Human iPSC-derived hepatocytes in 2D and 3D suspension culture for cryopreservation and in vitro toxicity studies. Reprod. Toxicol..

[18] Badenes S.M., Fernandes T.G., Rodrigues C.A.V., Diogo M.M., Cabral J.M.S. (2016). Microcarrier-based platforms for in vitro expansion and differentiation of human pluripotent stem cells in bioreactor culture systems. J. Biotechnol..

[19] Azarin S.M., Palecek S.P. (2010). Matrix revolutions: A trinity of defined substrates for long-term expansion of human ESCs. Cell Stem Cell.

[20] Gerke S., Taupitz J., Wiesemann C., Kopetzki C., Zimmermann H. (2020). Die klinische Anwendung von humanen induzierten pluripotenten Stammzellen.

[21] Meneghel J., Kilbride P., Morris G.J. (2020). Cryopreservation as a Key Element in the Successful Delivery of Cell-Based Therapies—A Review. Front. Med..

[22] Shibamiya A., Schulze E., Krauß D., Augustin C., Reinsch M., Schulze M.L., Steuck S., Mearini G., Mannhardt I., Schulze T. (2020). Cell Banking of hiPSCs: A Practical Guide to Cryopreservation and Quality Control in Basic Research. Curr. Protoc. Stem Cell Biol..

[23] Takahashi T., Hirsh A., Erbe E., Williams R.J. (1988). Mechanism of cryoprotection by extracellular polymeric solutes. Biophys. J..

[24] Mazur P. (1984). Freezing of living cells: Mechanisms and implications. Am. J. Physiol..

[25] Steeg R., Mueller S.C., Mah N., Holst B., Cabrera-Socorro A., Stacey G.N., de Sousa P.A., Courtney A., Zimmermann H. (2021). EBiSC best practice: How to ensure optimal generation, qualification, and distribution of iPSC lines. Stem Cell Rep..

[26] Heidemann R., Lünse S., Tran D., Zhang C. (2010). Characterization of cell-banking parameters for the cryopreservation of mammalian cell lines in 100-mL cryobags. Biotechnol. Prog..

[27] Spoerl S., Peter R., Krackhardt A.M. (2016). Cryopreservation in Closed Bag Systems as an Alternative to Clean Rooms for Preparations of Peripheral Blood Stem Cells. Biobanking and Cryopreservation of Stem Cells.

[28] Das A.T., Tenenbaum L., Berkhout B. (2016). Tet-On Systems For Doxycycline-inducible Gene Expression. Curr. Gene Ther..

[29] Ho S.-M., Hartley B.J., Tcw J., Beaumont M., Stafford K., Slesinger P.A., Brennand K.J. (2016). Rapid Ngn2-induction of excitatory neurons from hiPSC-derived neural progenitor cells. Methods.

[30] Hoang D.M., Pham P.T., Bach T.Q., Ngo A.T.L., Nguyen Q.T., Phan T.T.K., Nguyen G.H., Le P.T.T., van Hoang T., Forsyth N.R. (2022). Stem cell-based therapy for human diseases. Signal Transduct. Target. Ther..

[31] Bashor C.J., Hilton I.B., Bandukwala H., Smith D.M., Veiseh O. (2022). Engineering the next generation of cell-based therapeutics. Nat. Rev. Drug Discov..

[32] O’Shea O., Steeg R., Chapman C., Mackintosh P., Stacey G.N. (2020). Development and implementation of large-scale quality control for the European bank for induced Pluripotent Stem Cells. Stem Cell Res..

[33] Andrews P.W., Baker D., Benvinisty N., Miranda B., Bruce K., Brüstle O., Choi M., Choi Y.-M., Crook J.M., de Sousa P.A. (2015). Points to consider in the development of seed stocks of pluripotent stem cells for clinical applications: International Stem Cell Banking Initiative (ISCBI). Regen. Med..

[34] Manstein F., Halloin C., Zweigerdt R. (2019). Human Pluripotent Stem Cell Expansion in Stirred Tank Bioreactors. Methods Mol. Biol..

[35] Cohen P.J.R., Luquet E., Pletenka J., Leonard A., Warter E., Gurchenkov B., Carrere J., Rieu C., Hardouin J., Moncaubeig F. (2023). Engineering 3D micro-compartments for highly efficient and scale-independent expansion of human pluripotent stem cells in bioreactors. Biomaterials.

[36] Massie I., Selden C., Hodgson H., Fuller B., Gibbons S., Morris G.J. (2014). GMP cryopreservation of large volumes of cells for regenerative medicine: Active control of the freezing process. Tissue Eng. Part C Methods.

[37] Li T., Mai Q., Gao J., Zhou C. (2010). Cryopreservation of human embryonic stem cells with a new bulk vitrification method. Biol. Reprod..

[38] Li Y., Ma T. (2012). Bioprocessing of cryopreservation for large-scale banking of human pluripotent stem cells. Biores. Open Access.

[39] Hunt C.J. (2019). Technical Considerations in the Freezing, Low-Temperature Storage and Thawing of Stem Cells for Cellular Therapies. Transfus. Med. Hemother..

[40] Murray K.A., Gibson M.I. (2022). Chemical approaches to cryopreservation. Nat. Rev. Chem..

[41] Fuller B., Gonzalez-Molina J., Erro E., de Mendonca J., Chalmers S., Awan M., Poirier A., Selden C. (2017). Applications and optimization of cryopreservation technologies to cellular therapeutics. Cell Gene Ther. Insights.

[42] Watanabe K., Ueno M., Kamiya D., Nishiyama A., Matsumura M., Wataya T., Takahashi J.B., Nishikawa S., Muguruma K., Sasai Y. (2007). A ROCK inhibitor permits survival of dissociated human embryonic stem cells. Nat. Biotechnol..

[43] Li X., Meng G., Krawetz R., Liu S., Rancourt D.E. (2008). The ROCK inhibitor Y-27632 enhances the survival rate of human embryonic stem cells following cryopreservation. Stem Cells Dev..

[44] Miyazaki T., Nakatsuji N., Suemori H. (2014). Optimization of slow cooling cryopreservation for human pluripotent stem cells. Genesis.

[45] Ichikawa H., Yoshie S., Shirasawa S., Yokoyama T., Yue F., Tomotsune D., Sasaki K. (2011). Freeze-thawing single human embryonic stem cells induce e-cadherin and actin filament network disruption via g13 signaling. Cryo Lett..

[46] Vernardis S.I., Terzoudis K., Panoskaltsis N., Mantalaris A. (2017). Human embryonic and induced pluripotent stem cells maintain phenotype but alter their metabolism after exposure to ROCK inhibitor. Sci. Rep..

[47] Hunt C.J. (2011). Cryopreservation of Human Stem Cells for Clinical Application: A Review. Transfus. Med. Hemotherapy Off. Organ Dtsch. Ges. Transfusionsmedizin Immunhamatol..

[48] Yirme G., Amit M., Laevsky I., Osenberg S., Itskovitz-Eldor J. (2008). Establishing a dynamic process for the formation, propagation, and differentiation of human embryoid bodies. Stem Cells Dev..

[49] Baboo J., Kilbride P., Delahaye M., Milne S., Fonseca F., Blanco M., Meneghel J., Nancekievill A., Gaddum N., Morris G.J. (2019). The Impact of Varying Cooling and Thawing Rates on the Quality of Cryopreserved Human Peripheral Blood T Cells. Sci. Rep..

[50] Mazur P., Leibo S.P., Chu E.H. (1972). A two-factor hypothesis of freezing injury. Evidence from Chinese hamster tissue-culture cells. Exp. Cell Res..

[51] van Hoof D., Braam S.R., Dormeyer W., Ward-van Oostwaard D., Heck A.J.R., Krijgsveld J., Mummery C.L. (2008). Feeder-free monolayer cultures of human embryonic stem cells express an epithelial plasma membrane protein profile. Stem Cells.

[52] Rouhani F., Kumasaka N., de Brito M.C., Bradley A., Vallier L., Gaffney D. (2014). Genetic background drives transcriptional variation in human induced pluripotent stem cells. PLoS Genet..

[53] Katkov I.I., Kim M.S., Bajpai R., Altman Y.S., Mercola M., Loring J.F., Terskikh A.V., Snyder E.Y., Levine F. (2006). Cryopreservation by slow cooling with DMSO diminished production of Oct-4 pluripotency marker in human embryonic stem cells. Cryobiology.

[54] Xu X., Cowley S., Flaim C.J., James W., Seymour L., Cui Z. (2010). The roles of apoptotic pathways in the low recovery rate after cryopreservation of dissociated human embryonic stem cells. Biotechnol. Prog..

[55] Len J.S., Koh W.S.D., Tan S.-X. (2019). The roles of reactive oxygen species and antioxidants in cryopreservation. Biosci. Rep..

[56] Parmar M., Grealish S., Henchcliffe C. (2020). The future of stem cell therapies for Parkinson disease. Nat. Rev. Neurosci..

[57] Oh Y., Jang J. (2019). Directed Differentiation of Pluripotent Stem Cells by Transcription Factors. Mol. Cells.

[58] Shao Q., Yang T., Huang H., Alarmanazi F., Liu G. (2017). Uncoupling of UNC5C with Polymerized TUBB3 in Microtubules Mediates Netrin-1 Repulsion. J. Neurosci..

[59] Jovanov-Milošević N., Petanjek Z., Petrović D., Judaš M., Kostović I. (2010). Morphology, molecular phenotypes and distribution of neurons in developing human corpus callosum. Eur. J. Neurosci..

[60] Ortiz-Rodriguez J.M., Ortega-Ferrusola C., Gil M.C., Martín-Cano F.E., Gaitskell-Phillips G., Rodríguez-Martínez H., Hinrichs K., Álvarez-Barrientos A., Román Á., Peña F.J. (2019). Transcriptome analysis reveals that fertilization with cryopreserved sperm downregulates genes relevant for early embryo development in the horse. PLoS ONE.

[61] Paasch U., Sharma R.K., Gupta A.K., Grunewald S., Mascha E.J., Thomas A.J., Glander H.-J., Agarwal A. (2004). Cryopreservation and thawing is associated with varying extent of activation of apoptotic machinery in subsets of ejaculated human spermatozoa. Biol. Reprod..

[62] Pegg D.E. (2015). Principles of cryopreservation. Methods Mol. Biol..

